# Risk of gout among Taiwanese adults with ALDH-2 rs671 polymorphism according to BMI and alcohol intake

**DOI:** 10.1186/s13075-021-02497-9

**Published:** 2021-04-15

**Authors:** Yu-Ruey Liu, Disline Manli Tantoh, Chuan-Chao Lin, Chih-Hsuan Hsiao, Yung-Po Liaw

**Affiliations:** 1 Department of Emergency Medicine, Chung-Kang Branch, Cheng Ching Hospital, Taichung City, 407 Taiwan; 2 Department of Medical Imaging, Chung Shan Medical University Hospital, Taichung City, Taiwan; 3 Department of Public Health and Institute of Public Health, Chung Shan Medical University, No. 110 Sec. 1 Jianguo N. Road, Taichung City, 40201 Taiwan; 4 Department of Physical Medicine and Rehabilitation, Chung Shan Medical University Hospital, Taichung City, Taiwan; 5 School of Medicine, Chung Shan Medical University, Taichung City, Taiwan

**Keywords:** Alcohol drinking, BMI, ALDH2, rs671, Gout, Taiwan biobank

## Abstract

**Background:**

Gout stems from both modifiable and genetic sources. We evaluated the risk of gout among Taiwanese adults with aldehyde dehydrogenase-2 (ALDH2) rs671 single nucleotide polymorphism (SNP) according to body mass index (BMI) and alcohol drinking.

**Methods:**

We obtained information of 9253 individuals having no personal history of cancer from the Taiwan Biobank (2008–2016) and estimated the association between gout and independent variables (e.g., rs671, BMI, and alcohol drinking) using multiple logistic regression.

**Results:**

Alcohol drinking and abnormal BMI were associated with a higher risk of gout whereas the rs671 GA+AA genotype was associated with a lower risk. The odds ratios (ORs) and 95% confidence intervals (CIs) were 1.297 and 1.098–1.532 for alcohol drinking, 1.550 and 1.368–1.755 for abnormal BMI, and 0.887 and 0.800–0.984 for GA+AA. The interaction between BMI and alcohol on gout was significant for GG (*p*-value = 0.0102) and GA+AA (*p*-value = 0.0175). When we stratified genotypes by BMI, alcohol drinking was significantly associated with gout only among individuals with a normal BMI (OR; 95% CI = 1.533; 1.036–2.269 for GG and 2.109; 1.202–3.699 for GA+AA). Concerning the combination of BMI and alcohol drinking among participants stratified by genotypes (reference, GG genotype, normal BMI, and no alcohol drinking), the risk of gout was significantly higher in the following categories: GG, normal BMI, and alcohol drinking (OR, 95% CI = 1.929, 1.385–2.688); GG, abnormal BMI, and no alcohol drinking (OR, 95% CI, = 1.721, 1.442–2.052); GG, abnormal BMI, and alcohol drinking (OR, 95% CI = 1.941, 1.501–2.511); GA+AA, normal BMI, and alcohol drinking (OR, 95% CI = 1.971, 1.167–3.327); GA+AA, abnormal BMI, and no alcohol drinking (OR, 95% CI = 1.498, 1.256–1.586); and GA+AA, abnormal BMI, and alcohol drinking (OR, 95% CI = 1.545, 1.088–2.194).

**Conclusions:**

Alcohol and abnormal BMI were associated with a higher risk of gout, whereas the rs671 GA+AA genotype was associated with a lower risk. Noteworthy, BMI and alcohol had a significant interaction on gout risk. Stratified analyses revealed that alcohol drinking especially among normal-weight individuals might elevate the risk of gout irrespective of the genotype.

## Background

Gout is a metabolic disease that results from monosodium urate crystal deposits that are generally associated with high levels of urate serum [1, 2]. It is common worldwide and its incidence and prevalence are purportedly increasing [3]. Taiwan is among the top-tiered countries with a high prevalence of gout in the world [3, 4]. Data from Nutrition and Health Survey in Taiwan (NAHSIT) from 1993–1996 to 2005–2008 showed an increase in the prevalence of gout from 4.74 to 8.21% in men and 2.19 to 2.33% in women [5]. Moreover, a nationwide study revealed a prevalence of 6.24% and an incidence of 2.74 per 1000 person-years in 2010 [4].

Previous epidemiological studies identified numerous gout-related modifiable and non-modifiable factors, including but not limited to alcohol intake, BMI, cigarette smoking, sex, age, uric acid, and single nucleotide polymorphism [1, 2, 6–9]. BMI, a modifiable risk factor for gout [2, 3, 7, 8, 10, 11], is also related to well-established major risk factors for gout like hyperuricemia and alcohol consumption [3, 9, 12–15]. Alcohol is a proven key modifiable factor that has been specifically linked to higher incidence and prevalence of gout [2, 7, 8, 10, 11, 13, 16]. It is also a driving factor for hyperuricemia [17], a well-known precursor for gout [1, 2]. Alcohol could influence the risk of gout through its effect on uric acid [18–20]. ALDH2 rs671 attained genome-wide significance as a genetic locus for alcohol drinking [21].

ALDH2 is a vital enzyme in the metabolism of alcohol [22, 23]. The ALDH2 variant, rs671 is a missense SNP that impedes the enzymatic activity of the ALDH2, probably impacting metabolism that results in uric acid synthesis [24]. ALDH2 polymorphisms contribute not only to the metabolism of ethanol and acetaldehyde [25] but also impact predisposition to alcohol-related morbid conditions like hyperuricemia and gout among Asians [18, 19, 26–28]. The link between ALDH2 polymorphisms and serum urate was found to be mediated by alcohol intake among Han Chinese men [19]. ALDH2 rs671 is proven gout-related SNP [22, 29, 30].

Insights into interconnections between modifiable and genetic factors could aid in both the prevention and management of diseases. So far, a meta-analysis revealed that alcohol intake could modulate the link between BMI and ALDH2 rs671 among Koreans and Chinese [31]. Moreover, findings from GWAS suggest that BMI-associated alleles of rs671 are also linked to alcohol drinking behavior [25] and alcohol clearance [23]. The role of both BMI and alcohol drinking in the risk of gout according to ALDH2 rs671 genotypes has not been sufficiently investigated. As such, it is currently inconclusive whether the risk of gout varies based on the combination of these variables. In this study, we evaluated ALDH-2 rs671 polymorphism and the risk of gout according to two modifiable factors (BMI and alcohol intake) among Taiwanese adults.

## Materials and methods

### Data source and sample size

We used data from the Taiwan Biobank dataset (2008–2016). The Taiwan Biobank was established to build a data resource consisting of lifestyle and genetic data of a large cohort of Taiwanese adults aged 30 to 70 years. Data collection at Taiwan Biobank recruitment centers is done through questionnaires, biochemical, and physical examinations by well-trained personnel. Each participant signed a consent form prior to the collection of data. Initially, 9553 individuals filled the Taiwan Biobank questionnaires (containing data on alcohol drinking, sex, age, cigarette smoking, coffee/tea intake, exercise, and diet) and underwent both physical (e.g., weight, height, waist-hip ratio, and body fat) and biochemical tests (including genotyping, blood urea nitrogen, creatinine, HDL, LDL, and TG). However, 300 of them were ineligible for the study due to missing information. Hence, 9253 individuals were included in the final analyses. The Institutional Review Board of Cheng Ching General Hospital approved this study (HP200010).

### Description of variables

Gout cases were those who self-reported a clinical diagnosis of gout or those who were confirmed by biochemical tests to have serum urate levels ≥ 7 mg/dL (men) or ≥ 6 mg/dL (women). Alcohol drinking was defined as an intake of 150 cc of any alcoholic drink per week continuously for at least 6 months and at the time of data collection. No drinking was defined as drinking less than 150 cc of alcohol per week continuously for at least 6 months. Body mass index, calculated as weight (kg) divided by height squared (m^2^) was categorized into normal 18.5 ≤ BMI < 24 kg/m^2^ and abnormal 0 ≤ BMI < 18.5 and BMI ≥24  kg/m^2^. Waist-hip ratio (WHR), calculated as the ratio of waist to hip circumference was grouped into normal (< 0.9 for men and < 0.85 for women) and abnormal (≥ 0.9 for men and ≥ 0.85 for women). Body fat was classified as normal (< 25 for men and < 30% for women) or abnormal (≥ 25 and ≥ 30% for men and women, respectively). Tea consumption referred to drinking tea at least once per day. Exercise, cigarette smoking, coffee intake, and vegetarian diet were defined as previously elaborated [32–34]. Blood urea nitrogen levels above 20 mg/dL and creatinine levels (≥ 1.4 mg/dL in men and ≥ 1.2 mg/dL in women) were considered abnormal.

### Statistical analyses

The SNP (rs671) passed the quality control criteria (Hardy-Weinberg Equilibrium test *p*-value > 0.001), minor allele frequency ≥ 0.05, and call rate ≥ 95%. Chi-square test was used to estimate differences between categorical variables and the results were presented as *n* (%). The Student’s *t*-test was used to estimate differences between continuous variables and the results were presented as mean ± standard deviation (S.D). The interaction between BMI and alcohol drinking and the odds ratios for the association between the dependent (gout) and independent variables (rs671, BMI, alcohol drinking, etc.) were estimated using the multiple logistic regression analysis. In the regression models, we adjusted for covariates, including, sex, age, WHR, body fat, cigarette smoking, coffee intake, tea consumption, exercise, diet, blood urea nitrogen, creatinine, high-density lipoprotein cholesterol (HDL-C), low-density lipoprotein cholesterol (LDL-C), and triglycerides (TG). We used the dominant model for the SNP data because the enzyme activity in those with the rs671 GG genotype is higher compared to the AG and AA [24]. Moreover, a previous GWAS on gout and rs671 suggested that the dominant model is the model most likely to have higher statistical significance [22]. Data were managed and analyzed using PLINK v1.90 and SAS 9.4 software and the statistical threshold was set at *p*-value < 0.05 or Bonferroni correction value.

## Results

Table 1 presents the demographic features of cases (*n* = 2352) and non-cases (*n* = 6901) of gout. Individuals with and without gout were significantly different based on ALDH2 rs671 genotypes (*p*-value = 0.0122), alcohol drinking (*p*-value < 0.0001), and BMI (*p*-value < 0.0001).

**Table 1:** Demographic features of cases and non-cases of gout

Variables	No gout (*n* = 6901)	Gout (*n* = 2352)	*p*-value
Categorical variables	*n* (%)	*n* (%)	
ALDH2 rs671 genotype			
GG	3423 (49.60)	1237 (52.59)	0.0122
GA+AA	3478 (50.40)	1115 (47.41)	
Alcohol drinking			
No	6345 (91.94)	1966 (83.59)	< 0.0001
Yes	556 (8.06)	386 (16.41)	
Body mass index			
Normal (≥ 18.5 to <24Kg/m^2^)	3790 (54.92)	665 (28.27)	< 0.0001
Abnormal (< 18.5 and ≥ 24 Kg/m^2^)	3111 (45.08)	1687 (71.73)	
Sex			
Women	4163 (60.32)	773 (32.87)	< 0.0001
Men	2738 (39.68)	1579 (67.13)	
Age group (years)			
30–40	1803 (26.13)	612 (26.02)	0.0004
41–50	2001 (29.00)	584 (24.83)	
51–60	1944 (28.17)	718 (30.53)	
61–70	1153 (16.71)	438 (18.62)	
Waist-hip ratio			
Normal (men < 0.9; women < 0.85)	4048 (58.66)	960 (40.82)	< 0.0001
Abnormal (men ≥ 0.9; women ≥ 0.85)	2853 (41.34)	1392 (59.18)	
Body fat (%)			
Normal (men < 25; women < 30)	3953 (57.28)	987 (41.96)	< 0.0001
Abnormal (men ≥ 25; women ≥ 30)	2948 (42.72)	1365 (58.04)	
Cigarette smoking status			
Never	5564 (80.63)	1607 (68.32)	< 0.0001
Former	695 (10.07)	388 (16.50)	
Current	642 (9.30)	357 (15.18)	
Coffee consumption			
No	4613 (66.85)	1569 (66.71)	0.9036
Yes	2288 (33.15)	783 (33.29)	
Tea consumption			
No	4506 (65.29)	1322 (56.21)	< 0.0001
Yes	2395 (34.71)	1030 (43.79)	
Exercise			
No	3991 (57.83)	1370 (58.25)	0.7241
Yes	2910 (42.17)	982 (41.75)	
Diet status			
Non-vegetarian	6208 (89.96)	2174 (92.43)	< 0.0001
Former vegetarian	321 (4.65)	108 (4.59)	
Vegan	372 (5.39)	70 (2.98)	
Blood urea nitrogen (mg/dL)			
Normal (≤ 20)	6653 (96.41)	2187 (92.98)	< 0.0001
Abnormal (> 20)	248 (3.59)	165 (7.02)	
Creatinine (mg/dL)			
Normal (men < 1.4; women < 1.2)	6885 (99.77)	2303 (97.92)	< 0.0001
Abnormal (men ≥ 1.4; women ≥ 1.2)	16 (0.23)	49 (2.08)	
Continuous variables	Mean ± SD	Mean ± SD	
HDL-C (mg/dL)	54.96 ± 13.19	47.42 ± 11.17	< 0.0001
LDL-C (mg/dL)	118.70 ± 30.91	126.30 ± 32.91	< 0.0001
Triglycerides (mg/dL)	103.60 ± 75.71	155.10 ± 123.10	< 0.0001

*n* sample size, *ALDH2* aldehyde dehydrogenase 2, *SD* standard deviation, *HDL-C* high-density lipoprotein cholesterol, *LDC-C* low-density lipoprotein cholesterol

Table 2 shows the relationship of alcohol drinking, rs671 polymorphism, and BMI with gout. Alcohol drinking (reference, no drinking) and abnormal BMI (reference, normal BMI) were associated with a higher risk of gout while the GA+AA genotype (reference, GG) was associated with a lower risk. The ORs; 95% CIs; *p*-values were 1.297; 1.098–1.532; 0.0022 for alcohol drinking, 1.550; 1.368–1.755; < 0.0001 for abnormal BMI, and 0.887; 0.0240 for the GA+AA genotype. The interaction between BMI and alcohol on gout was significant (*p*-value = 0.006). However, the interaction of rs671 with alcohol and BMI was not significant (Table 2).

**Table 2:** Association of alcohol drinking, BMI, and ALDH2 rs671 polymorphism with gout

Variables	OR	95% CI	*p*-value
Alcohol drinking (ref, no)			
Yes	1.297	1.098–1.532	0.0022
Body mass index (ref, normal)			
Abnormal	1.550	1.368–1.755	< 0.0001
ALDH2 rs671 genotype (ref, GG)			
GA+AA	0.887	0.800–0.984	0.0240
Sex (ref, women)			
Men	2.363	2.068–2.700	< 0.0001
Age group (ref, 30–40 years)			
41–50	0.710	0.614–0.821	< 0.0001
51–60	0.847	0.731–0.981	0.0272
61–70	0.871	0.733–1.034	0.1155
Waist-hip ratio (ref, normal)			
Abnormal	1.358	1.212–1.522	< 0.0001
Body fat (ref, normal)			
Abnormal	1.445	1.272–1.640	< 0.0001
Cigarette smoking status (ref, never)			
Former	0.939	0.800–1.102	0.4409
Current	0.791	0.667–0.939	0.0073
Coffee consumption (ref, no)			
Yes	1.066	0.955–1.190	0.2536
Tea consumption (ref, no)			
Yes	1.223	1.099–1.361	0.0002
Exercise (ref, no)			
Yes	1.035	0.927–1.157	0.5380
Diet status (ref, non-vegetarian)			
Former vegetarian	1.013	0.792–1.296	0.9169
Vegan	0.656	0.497–0.867	0.0030
Blood urea nitrogen (ref, normal)			
Abnormal	1.420	1.118–1.803	0.0041
Creatinine (ref, normal)			
Abnormal	5.320	2.846–9.945	< 0.0001
HDL-C	0.980	0.975–0.985	< 0.0001
LDL-C	1.006	1.005–1.008	< 0.0001
Triglycerides	1.003	1.002–1.004	< 0.0001

Interaction between BMI and alcohol drinking (*p*-value = 0.0006)

*BMI* body mass index, *OR* odds ratio, *CI* confidence interval, *ref* reference, *ALDH2* aldehyde dehydrogenase 2, *HDL-C* high-density lipoprotein cholesterol, *LDC-C* low-density lipoprotein cholesterol

Table 3 shows the association of alcohol drinking and BMI with gout stratified by rs671 genotypes (GG and GA+AA). Both BMI and alcohol drinking were associated with a higher risk of gout. For alcohol, the association was significant in only the GG category (OR = 1.289; 95% CI = 1.048–1.586; *p*-value = 0.162). However, for BMI, the association was significant in both the GG (OR = 1.584; 95% CI = 1.332–1.883; *p*-value < 0.0001) and GA+AA (OR = 1.518; 95% CI = 1.268–1.818; *p*-value < 0.0001) categories. The interaction between BMI and alcohol on gout was significant for both GG (*p*-value = 0.0102) and GA+AA (*p*-value = 0.0175).

**Table 3:** Association of alcohol drinking and BMI with gout stratified by ALDH2 rs671 genotypes

Variables	GG (*n* = 4660)			GA+AA (*n* = 4593)		
	OR	95% CI	*p*-value	OR	95% CI	*p*-value
Alcohol drinking (ref, no)						
Yes	1.289	1.048–1.586	0.0162	1.273	0.950–1.706	0.1062
Body mass index (ref, normal)						
Abnormal	1.584	1.332–1.883	< 0.0001	1.518	1.268–1.818	< 0.0001
Sex (ref, women)						
Men	2.322	1.924–2.802	< 0.0001	2.378	1.966–2.876	< 0.0001
Age group (ref, 30–40 years)						
41–50	0.633	0.516–0.776	< 0.0001	0.800	0.649–0.986	0.0362
51–60	0.798	0.649–0.981	0.0318	0.899	0.727–1.111	0.0248
61–70	0.917	0.722–1.164	0.4750	0.821	0.640–1.054	0.1215
Waist-hip ratio (ref, normal)						
Abnormal	1.301	1.109–1.526	0.0012	1.419	1.205–1.670	< 0.0001
Body fat (ref, normal)						
Abnormal	1.469	1.230–1.755	< 0.0001	1.414	1.178–1.698	0.0002
Cigarette smoking status (ref, never)						
Former	1.081	0.865–1.351	0.4948	0.811	0.644–1.023	0.0769
Current	0.916	0.720–1.165	0.4745	0.698	0.546–0.892	0.0041
Coffee consumption (ref, no)						
Yes	1.142	0.981–1.329	0.0878	0.991	0.845–1.163	0.9141
Tea consumption (ref, no)						
Yes	1.195	1.029–1.388	0.0197	1.263	1.084–1.472	0.0028
Exercise (ref, no)						
Yes	0.996	0.854–1.162	0.9594	1.084	0.924–1.272	0.3232
Diet status (ref, non-vegetarian)						
Former vegetarian	1.172	0.828–1.660	0.3704	0.869	0.612–1.234	0.4317
Vegan	0.647	0.437–0.959	0.0299	0.657	0.443–0.975	0.0370
Blood urea nitrogen (ref, normal)						
Abnormal	1.268	0.900–1.786	0.1744	1.558	1.113–2.182	0.0098
Creatinine (ref, normal)						
Abnormal	6.216	2.738–14.111	< 0.0001	4.418	1.630–11.972	0.0035
HDL-C	0.981	0.974–0.988	< 0.0001	0.979	0.971–0.987	< 0.0001
LDL-C	1.007	1.005–1.009	< 0.0001	1.006	1.003–1.008	< 0.0001
Triglycerides	1.003	1.002–1.004	< 0.0001	1.003	1.002–1.004	< 0.0001

Interaction between BMI and alcohol drinking (*p*-value = 0.0102 and 0.0175 for the GG and GA+AA group, respectively)

*BMI* body mass index, *OR* odds ratio, *CI* confidence interval, *ref* reference, *ALDH2* Aldehyde dehydrogenase 2, *HDL-C* high-density lipoprotein cholesterol, *LDC-C* low-density lipoprotein cholesterol

Tables 4 and 5 illustrate the association between alcohol drinking and gout among participants with ALDH2 rs671 GG and GA+AA stratified by BMI. Alcohol drinking was significantly associated with gout only among individuals with a normal BMI. This results were observed for both GG: OR; 95% CI; *p*-value = 1.533; 1.036–2.269; 0.0325 (Table 4) and GA+AA: OR; 95% CI; *p*-value = 2.109; 1.202–3.699; 0.0092 (Table 5).

**Table 4:** Association between alcohol drinking and gout among participants with the ALDH2 rs671 GG genotype stratified by BMI

Variables	GG genotype (*n* = 4660)					
	Normal (*n* = 2239)			Abnormal (*n* = 2421)		
	OR	95% CI	*P*-value	OR	95% CI	*P*-value
Alcohol drinking (ref: no)						
yes	1.533	1.036-2.269	0.0325	1.205	0.945-1.538	0.1331
Sex (ref: women)						
men	2.742	1.996-3.765	<0.0001	2.116	1.671-2.681	<0.0001
Age group (ref: 30-40 years)						
41-50	0.711	0.492-1.028	0.0697	0.578	0.450-0.742	<0.0001
51-60	1.064	0.740-1.531	0.7370	0.638	0.495-0.823	0.0005
61-70	0.906	0.591-1.391	0.6533	0.850	0.634-1.141	0.2792
Waist-hip ratio (ref: normal)						
abnormal	1.647	1.246-2.177	0.0005	1.166	0.959-1.418	0.1236
Body fat (ref: normal)						
abnormal	1.244	0.900-1.718	0.1861	1.595	1.282-1.985	<0.0001
Cigarette smoking status (ref: never)						
former	1.098	0.720-1.675	0.6640	1.121	0.861-1.459	0.3961
current	1.032	0.666-1.600	0.8881	0.839	0.629-1.120	0.2344
Coffee consumption (ref: no)						
yes	1.235	0.946-1.611	0.1207	1.112	0.922-1.341	0.2660
Tea consumption (ref: no)						
yes	1.237	0.946-1.618	0.1204	1.181	0.985-1.416	0.0721
Exercise (ref: no)						
yes	1.303	0.995-1.706	0.0545	0.860	0.712-1.039	0.1180
Diet status (ref: non-vegetarian)						
former vegetarian	0.770	0.386-1.539	0.4603	1.480	0.966-2.268	0.0716
vegan	0.689	0.383-1.238	0.2124	0.554	0.323-0.950	0.0318
Blood urea nitrogen (ref: normal)						
abnormal	0.865	0.441-1.694	0.6721	1.544	1.028-2.319	0.0363
Creatinine (ref: normal)						
abnormal	19.851	3.691-106.761	0.0005	3.885	1.542-9.790	0.0040
HDL-C	0.980	0.969-0.991	0.0006	0.981	0.973-0.990	<0.0001
LDL-C	1.010	1.006-1.014	<0.0001	1.005	1.002-1.008	0.0004
Triglycerides	1.004	1.002-1.006	<0.0001	1.002	1.001-1.003	<0.0001

*BMI* body mass index, *OR* odds ratio, *ref* reference, *ALDH2* aldehyde dehydrogenase 2, *HDL-C* high density lipoprotein cholesterol, *LDC-C* low density lipoprotein cholesterol

**Table 5:** Association between alcohol drinking and gout among participants with the ALDH2 rs671 GA+AA genotype stratified by BMI

Variables	GA+AA genotype (n = 4593)					
	Normal (*n* = 2216)			Abnormal (*n* = 2377)		
	OR	95% CI	*p*-value	OR	95% CI	*p*-value
Alcohol drinking (ref, no)						
Yes	2.109	1.202–3.699	0.0092	1.108	0.789–1.557	0.5525
Sex (ref, women)						
Men	2.561	1.854–3.539	< 0.0001	2.190	1.724–2.780	< 0.0001
Age group (ref, 30–40 years)						
41–50	1.119	0.766–1.635	0.5601	0.664	0.514–0.857	0.0017
51–60	1.329	0.905–1.951	0.1469	0.743	0.572–0.965	0.0260
61–70	1.536	0.991–2.382	0.0552	0.589	0.433–0.803	0.0008
Waist-hip ratio (ref, normal)						
Abnormal	1.410	1.063–1.869	0.0171	1.441	1.176–1.766	0.0004
Body fat (ref, normal)						
Abnormal	1.331	0.955–1.856	0.0913	1.392	1.113–1.742	0.0038
Cigarette smoking status (ref, never)						
Former	0.527	0.326–0.851	0.0088	0.950	0.725–1.243	0.7065
Current	0.890	0.580–1.365	0.5936	0.591	0.438–0.798	0.0006
Coffee consumption (ref, no)						
Yes	0.955	0.720–1.268	0.7516	0.996	0.820–1.211	0.9712
Tea consumption (ref, no)						
Yes	1.562	1.193–2.046	0.0012	1.150	0.953–1.387	0.1454
Exercise (ref, no)						
Yes	1.155	0.873–1.527	0.3127	1.024	0.841–1.248	0.8108
Diet status (ref, non-vegetarian)						
Former vegetarian	0.443	0.200–0.979	0.0442	1.090	0.721–1.650	0.6821
Vegan	0.492	0.247–0.980	0.0437	0.765	0.466–1.254	0.2876
Blood urea nitrogen (ref, normal)						
Abnormal	2.231	1.313–3.791	0.0030	1.217	0.792–1.871	0.3696
Creatinine (ref, normal)						
Abnormal	1.715	0.407–7.234	0.4626	10.705	2.082–55.046	0.0045
HDL-C	0.979	0.967–0.991	0.0007	0.979	0.969–0.989	< 0.0001
LDL-C	1.006	1.002–1.010	0.0053	1.006	1.003–1.009	0.0002
Triglycerides	1.004	1.002–1.005	< 0.0001	1.003	1.002–1.004	< 0.0001

*BMI* body mass index, *OR* odds ratio, *CI* confidence interval, *ref* reference, *ALDH2* aldehyde dehydrogenase 2, *HDL-C* high-density lipoprotein cholesterol, *LDC-C* low-density lipoprotein cholesterol

Table 6 shows the risk of gout in relation to the combination of BMI and alcohol drinking among participants stratified by ALDH2 rs671 genotypes. Compared to the reference category (no alcohol drinking and normal BMI), the risk of gout was significantly higher for both GG and GA+AA. For the GG category, the ORs (95% CI; *p*-value) were 1.851 (1.316–2.603; 0.0004) for normal BMI and alcohol drinking, 1.727 (1.433–2.080; < 0.0001) for abnormal BMI and no alcohol drinking, and 1.913 (1.451–2.523; < 0.0001) for abnormal BMI and alcohol drinking. For the GA+AA category, the OR (95% CI; *p*-value) were 2.212 (1.302–3.757; 0.0033) for normal BMI and alcohol drinking, 1.592 (1.323–1.916; < 0.0001) for abnormal BMI and no alcohol drinking, and 1.675 (1.166–2.407; 0.0053) for abnormal BMI and alcohol drinking.

**Table 6:** Risk of gout in relation to the combination of BMI and alcohol drinking among participants stratified by ALDH2 rs671 genotypes

Variables	GG (n = 4660)			GA+AA (n = 4593)		
	OR	95% CI	*p*-value	OR	95% CI	*p*-value
BMI and alcohol drinking (ref, normal BMI and no alcohol drinking)						
Normal BMI and alcohol drinking	1.851	1.316–2.603	0.0004	2.212	1.302–3.757	0.0033
Abnormal BMI and no alcohol drinking	1.727	1.433–2.080	< 0.0001	1.592	1.323–1.916	< 0.0001
Abnormal BMI and alcohol drinking	1.913	1.451–2.523	< 0.0001	1.675	1.166–2.407	0.0053
Sex (ref, women)						
Men	2.312	1.915–2.790	< 0.0001	2.377	1.965–2.876	< 0.0001
Age group (ref, 30–40 years)						
41–50	0.633	0.516–0.777	< 0.0001	0.798	0.648–0.983	0.0343
51–60	0.798	0.649–0.981	0.0320	0.904	0.731–1.118	0.3501
61–70	0.914	0.720–1.161	0.4632	0.820	0.639–1.052	0.1189
Waist-hip ratio (ref, normal)						
Abnormal	1.304	1.111–1.529	0.0011	1.426	1.211–1.679	< 0.0001
Body fat (ref, normal)						
Abnormal	1.479	1.237–1.767	< 0.0001	1.415	1.178–1.699	0.0002
Cigarette smoking status (ref, never)						
Former	1.078	0.863–1.347	0.5082	0.810	0.643–1.021	0.0745
Current	0.903	0.710–1.149	0.4079	0.692	0.541–0.884	0.0033
Coffee consumption (ref, no)						
Yes	1.131	0.971–1.317	0.1130	0.984	0.839–1.155	0.8477
Tea consumption (ref, no)						
Yes	1.192	1.026–1.384	0.0215	1.266	1.086–1.476	0.0026
Exercise (ref, no)						
Yes	1.001	0.858–1.168	0.9904	1.084	0.924–1.272	0.3235
Diet status (ref, non-vegetarian)						
Former vegetarian	1.155	0.815–1.636	0.4186	0.869	0.612–1.234	0.4333
Vegan	0.641	0.433–0.950	0.0267	0.663	0.447–0.985	0.0417
Blood urea nitrogen (ref, normal)						
Abnormal	1.279	0.909–1.801	0.1576	1.561	1.114–2.187	0.0097
Creatinine (ref, normal)						
Abnormal	6.192	2.726–14.067	< 0.0001	4.449	1.639–12.076	0.0034
HDL-C	0.980	0.973–0.987	< 0.0001	0.979	0.972–0.987	< 0.0001
LDL-C	1.007	1.005–1.009	< 0.0001	1.006	1.003–1.008	< 0.0001
Triglycerides	1.003	1.002–1.004	< 0.0001	1.003	1.002–1.004	< 0.0001

*BMI* body mass index, *OR* odds ratio, *CI* confidence interval, *ref* reference, *ALDH2* Aldehyde dehydrogenase 2, *HDL-C* high-density lipoprotein cholesterol, *LDC-C* low-density lipoprotein cholesterol

Table 7 displays the risk of gout in relation to the combination of BMI and alcohol drinking among participants stratified by ALDH2 rs671 genotypes. Compared to the reference category (GG genotype, normal BMI, and no alcohol drinking), the risk of gout was significantly higher for all but one category (GA+AA, normal BMI, and no alcohol drinking). The OR (95% CI; *p*-value) was 1.929 (1.385–2.688; 0.0001) for GG, normal BMI, and alcohol drinking; 1.721 (1.442–2.052; < 0.0001) for GG, abnormal BMI, and no alcohol drinking; 1.941 (1.501–2.511; < 0.0001) for GG, abnormal BMI, and alcohol drinking; 0.937 (0.779–1.126; 0.4862) for GA+AA, normal BMI, and no alcohol drinking; 1.971 (1.167–3.327; 0.0111) for GA+AA, normal BMI, and alcohol drinking; 1.498 (1.1256–1.786; < 0.0001) for GA+AA, abnormal BMI, and no alcohol drinking; and 1.545 (1.088–2.194; 0.0150) for GA+AA, abnormal BMI, and alcohol drinking. Some covariates that were consistently associated with gout (Tables 2, 3, 4, 5, 6, and 7) included sex (high risk in men compared to women), HDL-C (lower risk), LDL-C (higher risk), and TG (higher risk).

**Table 7:** Risk of gout in relation to the combination of BMI, alcohol drinking, and ALDH2 rs671 polymorphism

Variables	OR	95% CI	*p*-value
ALDH2 rs671 genotypes, BMI, and alcohol drinking (ref, GG, normal BMI, no alcohol drinking)			
GG, normal BMI, and alcohol drinking	1.929	1.385–2.688	0.0001
GG, abnormal BMI, and no alcohol drinking	1.721	1.442–2.052	< 0.0001
GG, abnormal BMI, and alcohol drinking	1.941	1.501–2.511	< 0.0001
GA+AA, normal BMI, and no alcohol drinking	0.937	0.779–1.126	0.4862
GA+AA, normal BMI, and alcohol drinking	1.971	1.167–3.327	0.0111
GA+AA, abnormal BMI, and no alcohol drinking	1.498	1.256–1.786	< 0.0001
GA+AA, abnormal BMI, and alcohol drinking	1.545	1.088–2.194	0.0150
Sex (ref, women)			
Men	2.355	2.061–2.692	< 0.0001
Age group (ref, 30–40 years)			
41–50	0.709	0.613–0.820	< 0.0001
51–60	0.849	0.732–0.984	0.0297
61–70	0.869	0.731–1.032	0.1083
Waist-hip ratio (ref, normal)			
Abnormal	1.362	1.216–1.527	< 0.0001
Body fat (ref, normal)			
Abnormal	1.450	1.276–1.647	< 0.0001
Cigarette smoking status (ref, never)			
Former	0.937	0.799–1.100	0.4268
Current	0.782	0.659–0.928	0.0049
Coffee consumption (ref, no)			
Yes	1.058	0.948–1.180	0.3177
Tea consumption (ref, no)			
Yes	1.223	1.099–1.360	0.0002
Exercise (ref, no)			
Yes	1.039	0.930–1.160	0.5007
Diet status (ref, non-vegetarian)			
Former vegetarian	1.006	0.787–1.286	0.9623
Vegan	0.656	0.497–0.867	0.0030
Blood urea nitrogen (ref, normal)			
Abnormal	1.426	1.123–1.812	0.0036
Creatinine (ref, normal)			
Abnormal	5.324	2.845–9.962	< 0.0001
HDL-C	0.980	0.975–0.985	< 0.0001
LDL-C	1.006	1.005–1.008	< 0.0001
Triglycerides	1.003	1.002–1.004	< 0.0001

*BMI* body mass index, *OR* odds ratio, *CI* confidence interval, *ref* reference, *ALDH2* Aldehyde dehydrogenase 2, *HDL-C* high-density lipoprotein cholesterol, *LDC-C* low-density lipoprotein cholesterol

## Discussion

In the present study, the rs671 GA+AA genotype was associated with a lower risk of gout, while alcohol and abnormal BMI were associated with a higher risk. Of note, BMI and alcohol had a significant interaction on gout risk among individuals with GG and GA+AA. However, there was no significant interaction of rs671 with either BMI or alcohol drinking. Stratified analyses revealed that alcohol drinking especially among normal-weight individuals could confer susceptibility to gout, irrespective of genotype. These findings confirm the major role of alcohol consumption in the risk of gout. However, we cannot state the precise underlying biological mechanisms. Similar to our results, significant interactions between BMI and alcohol on hyperuricemia have been documented [17, 35]. Based on their findings, Shiraishi and Une advised obese people to reduce the amount of alcohol they consume [35].

Many past studies reported significant associations between gout and rs671 [22, 29, 30, 36]. This variant was described as a real gout-SNP [22, 29, 30]. The A allele of the rs671 has been linked to reduced susceptibility to gout [22]. ALDH2 rs671 also demonstrated the strongest GWA significance for alcohol drinking [21]. It was found to be related to alcohol drinking habits and alcohol flushing responses in Asians [25, 37]. Rapid metabolism of acetaldehyde and ethanol associated with a homozygous ALDH2 rs671 genotype was linked to higher levels of UA in Japanese alcoholic men [26]. The relationship between gout and rs671 could in part be accounted for by alcohol drinking [22].

Previous studies on the risk of gout based on alcohol consumption showed conflicting findings. Most pioneer epidemiological research reported no association, probably because of a relatively small number of gout cases and failure to adjust for vital confounders [38–40]. Nonetheless, subsequent studies with higher gout cases showed significant associations [13, 16]. A potential explanatory mechanism implicated in the relationship between gout and alcohol is that it enhances uric acid production and the hepatic breakdown of adenosine triphosphate (ATP) [41]. Moreover, alcoholic drinks like beer are rich in purine, which is associated with high levels of uric acid [42].

Evidence from a study using the UK biobank data suggested that genetic polymorphisms have a strong effect on gout regardless of BMI [43]. ALDH2 rs671 attained a significant genome-wide association for BMI [31] and was reported as the only locus having a significant independent association with BMI [31]. Numerous prospective studies on Asians, Europeans, and Americans suggested that BMI is positively related to the odds of gout and this relationship is possibly mediated by several factors [8, 9, 39, 43–52]. However, there were also reports of no significant relationship between BMI and gout [40]. The role of BMI in gout pathogenesis could be elucidated based on how leptin responds to inflammation related to monosodium urate crystals [53, 54]. BMI could also cause gout through its effect on serum urate [52, 55], potentially through insulinemia which affects renal reabsorption and uric acid clearance [56–59].

Previous studies also had similar findings on the risk of gout pertaining to sex, cigarette smoking, lipoproteins, and other variables [6, 7, 60, 61].

The current study is limited in that the gout population in this study may not be representative of gout patients in the general population. This is because about 33% of gout cases were women. This percentage appears high given that the prevalence of gout in Taiwanese men is about 4 times higher than that in women. Moreover, we defined cases as those who self-reported a clinical diagnosis of gout or those with uric acid levels ≥ 7 mg/dL (men) or ≥ 6 mg/dL (women). However, there was no information regarding patients on effective ULT and so the results are possibly not generalizable. In addition, the cohort is 25% gout cases and is thus closer to a case-control cohort than a general population sample. Another limitation of our study is that we could not clearly explain the precise biological mechanisms underlying the reported relationships.

## Conclusion

Alcohol and abnormal BMI were associated with a higher risk of gout, while the rs671 GA+AA genotype was associated with a lower risk. Of note, BMI and alcohol had a significant interaction on gout risk among individuals with GG and GA+AA. Stratified analyses revealed that alcohol drinking, especially among normal-weight individuals confers a great risk of gout irrespective of genotype. These findings confirm the major role of alcohol consumption on gout and so both normal weight and abnormal weight individuals are advised to reduce the amount of alcohol they consume. Reducing the amount of alcohol intake could play a great role in public health as it might mitigate the risk of gout.

## Data Availability

The data that support the findings of this study are available from Taiwan Biobank but restrictions apply to the availability of these data, which were used under license for the current study and so are not publicly available. Data are however available from Professor Yung Po Liaw (email address, Liawyp@csmu.edu.tw; tel, + 886424730022 ext. 12102) upon reasonable request and with permission of Taiwan Biobank.

## Abbreviations

BMI: Body mass index
OR: Odds ratio
ref: Reference
ALDH2: Aldehyde dehydrogenase 2
HDL-C: High-density lipoprotein cholesterol
LDC-C: Low-density lipoprotein cholesterol
